# Coronary heart disease and tuberculosis: an unnoticed syndemia. Review of literature and management proposal

**DOI:** 10.47487/apcyccv.v5i2.375

**Published:** 2024-06-24

**Authors:** Mauricio Andrés Murillo Moreno, Laura Valentina López Gutiérrez, Eric Edward Vinck, Gustavo Roncancio Villamil, Catalina Gallego Muñoz, Clara Inés Saldarriaga Giraldo

**Affiliations:** 1 Departamento de Medicina Interna, Universidad CES, Medellín, Colombia. Universidad CES Departamento de Medicina Interna Universidad CES Medellín Colombia; 2 Departamento de Cardiología, Clínica Cardio VID, Medellín, Colombia. Departamento de Cardiología Clínica Cardio VID Medellín Colombia; 3 Departamento de Cirugía Cardiovascular, Clínica Cardio VID, Medellín, Colombia. Departamento de Cirugía Cardiovascular Clínica Cardio VID Medellín Colombia; 4 Departamento de Enfermedades Infecciosas, Clínica Cardio VID, Medellín, Colombia. Departamento de Enfermedades Infecciosas Clínica Cardio VID Medellín Colombia

**Keywords:** Tuberculosis, Heart failure, Coronary Disease, Myocardial Infarction, Tuberculosis, Insuficiencia Cardiaca, Enfermedad Coronaria, Infarto del Miocardio

## Abstract

Tuberculosis is an increasing disease that affects about one-third of the global population. In line with the rise of tuberculosis, cardiovascular disease has shown a similar trend, with ischemic coronary heart disease becoming the leading cause of death worldwide. Based on the literature, a relationship can be drawn between tuberculosis and ischemic coronary heart disease through their shared multiple risk factors and a possible pathophysiological substrate linking them. The presentation of these two conditions reported so far is varied: it has been found as the onset of acute coronary syndrome in patients with active tuberculosis, the progressive development of coronary atherosclerosis in patients with latent tuberculosis, among others. Given this possible link and the progressive increase in their incidence rates, we can assert that we are facing an unnoticed syndemic, with their concurrent management posing a challenge due to significant pharmacological interactions. The purpose of this review is to clarify this possible link, propose an approach for diagnosis, and provide a treatment algorithm for the entire spectrum of coronary disease coexisting with tuberculosis according to the current available literature.

## RESUMEN

La tuberculosis es una enfermedad que viene en aumento y que afecta cerca de un tercio de la población mundial. En consonancia con el aumento de la tuberculosis, la enfermedad cardiovascular ha tenido un comportamiento similar, de este grupo, la cardiopatía coronaria isquémica se ha convertido en la principal causa de muerte en todo el mundo. Se podría extraer, en base a la literatura, una relación entre la tuberculosis y la cardiopatía coronaria isquémica a través de que comparten múltiples factores de riesgo en común y desde un posible sustrato fisiopatológico que las vincula. La forma conjunta de presentación de estas dos entidades reportada hasta el momento es variada: se ha encontrado como debut de un síndrome coronario agudo en pacientes con tuberculosis activa, el desarrollo progresivo de aterosclerosis coronaria en pacientes con tuberculosis latente, entre otras. Dado este posible vínculo y el aumento progresivo de sus tasas de incidencia podemos afirmar que estamos ante una sindemia inadvertida, siendo su manejo conjunto un desafío por las grandes interacciones farmacológicas. El propósito de esta revisión es esclarecer este posible vínculo, plantear un enfoque para el diagnóstico, así como suministrar un algoritmo de tratamiento de todo el espectro de la enfermedad coronaria que coexiste con la tuberculosis de acuerdo con la literatura actual disponible.

## Introduction

Tuberculosis (TB) is a granulomatous disease caused by acid-fast, gram-positive bacilli of the genus *Mycobacterium*^1^. Although primarily a pulmonary disease, TB can affect any organ in the body, including the heart ^2^. This microorganism infects approximately one-third of the world’s population ^3^, and in certain Latin American countries like Colombia, the reported incidence of pulmonary tuberculosis has increased, as described in the epidemiology section ^4^^,^^5^. Conversely, cardiovascular diseases are the leading cause of death globally ^6^. In Colombia ^7^, coronary artery disease has been the predominant cause of cardiovascular-related deaths reported from 1998 to 2011 ^8^. 

It has recently been shown that people with tuberculosis have a higher risk of cardiovascular diseases, demonstrating that these two conditions are interrelated ^6^. This relationship may be explained by the fact that they share similar risk factors and due to the description of a possible pathophysiological substrate that links them ^9^^-^^12^, as will be discussed later.

The combined treatment of both conditions presents a challenge, not only because they require long-term pharmacological management but also because polypharmacy can lead to undesirable drug interactions, potentially resulting in severe and unexpected outcomes. However, the current medical literature lacks information on how to approach diagnosis and treatment in patients with active tuberculosis and part of the cardiovascular disease spectrum, such as ischemic coronary artery disease. There are no guidelines to maximize appropriate combined treatment or address cases requiring coronary surgical intervention in the context of active tuberculosis. Therefore, we conducted a review of the relationship between these two conditions and proposed a diagnostic and management approach based on the available literature. With this, we aim to reduce the knowledge gap in the management of coronary artery disease and heart failure coexisting with tuberculosis. 

### Epidemiology

In humans, tuberculosis is primarily caused by *Mycobacterium tuberculosis*, which predominantly affects the lungs, causing pulmonary tuberculosis ^1^. This microorganism infects about one-third of the global population, with a 10% lifetime risk of developing the disease ^3^. According to the World Health Organization (WHO), in 2021, there were 6.4 million cases globally and a total of 1.6 million deaths due to this disease ^13^. In Latin American countries like Colombia, the situation is equally alarming. In 2021, the incidence rate of pulmonary tuberculosis was reported to be 22.36 per 100,000 inhabitants, an increase from 21.35 per 100,000 inhabitants in 2020 ^4^. In Peru, the morbidity rate of tuberculosis increased from 84.40 per 100,000 inhabitants to 91.12 per 100,000 inhabitants from 2021 to 2022, respectively ^5^. Cardiovascular disease has shown a similar trend, with ischemic heart disease currently being the leading cause of death in Colombia ^7^, contributing to 56.3% of deaths from cardiovascular disease reported from 1998 to 2011 ^7^. These data highlight the high probability of the concurrent presentation of tuberculosis and coronary artery disease in any of their manifestations. 

### Concurrent presentation 

Although we do not have data demonstrating their combined incidence, there are various case reports in the literature that could link them ^14^^-^^25^. According to these reports, the onset of both the infectious disease and cardiovascular events is varied and can present in the context of coronary artery bypass grafting (CABG) as sternal wound infection due to tuberculosis ^16^^,^^18^, tuberculous pleural effusion ^15^, tuberculous pericardial effusion ^17^, tuberculous constrictive pericarditis ^23^, or pulmonary tuberculosis ^19^. It can also present as sudden cardiac death in the context of myocardial infarction due to tuberculous coronary arteritis ^21^, infarction associated with coronary aneurysm due to tuberculous arteritis ^25^, and acute atherosclerotic myocardial infarction associated with latent tuberculosis ^24^. Table 1 shows the most important clinical characteristics of these previously reported cases. 

**Table 1:** Case reports of tuberculosis associated with various presentations of coronary disease

Cases	Gender and Age	Comorbidities	Symptoms	TB diagnosis	Cardiac Disease/}Procedure	Anti-TB Treatment	Surgical Treatment	Outcome
TB in sternal wound post-CABG ^16^	F, 57 years	Hypertension, dyslipidemia, T2DM, CKD on RRT, CABG 8 months ago for three-vessel coronary disease	Nodular lesions with erythema and purulent discharge from the sternal surgical wound	Ziehl-Neelsen stain positive for AFB+ PCR positive for Mycobacterium tuberculosis from sternal secretion	Three-vessel coronary disease/CABG	Isoniazid + Pyrazinamide + Levofloxacin + Ethambutol	None	Three months of follow-up without recurrence
Eleven patients with TB in sternal wound post-cardiac surgery ^14^	M: 8 patients (72.7%), F: 3 patients (27.3%), mean age: 58.6 years	T2DM, hypertension, dyslipidemia, and CKD on RRT	Purulent discharge (58%), abscess (33.3%), subcutaneous tract (33.3%), local pain (16.6%), fever (16.7%), sternal swelling (83%), and sternal mass (83%)	Culture (85.7%), Histopathology (64.3%), Ziehl-Neelsen stain (21.4%), PCR (71%) from sternal secretion	CABG (57.8%), unspecified surgery (28.5%), aortic valve replacement (7.1%), mitral valve replacement (7.1%), Bentall surgery (7.1%)	Not specified	Debridement (46.2%), extensive resection with wall reconstruction (38.5%)	Survival in all patients, 3 had 1-2 recurrences
Tuberculous pleural effusion post-CABG ^13^	M, 62 years	Not specified	Chest pain and exertional dyspnea	Pleural fluid culture and pleural histopathology	Non-ST elevation myocardial infarction/three-vessel coronary disease	Not specified	None	Six months of follow-up without complications
Tuberculous pericardial effusion post-CABG ^15^	M, 47 years	Not specified	Exertional dyspnea	Pericardial histopathology	Late tuberculous pericardial effusion post-CABG	HRZE + Prednisolone	Pericardial fluid drainage	Six months of follow-up without recurrence
Fatal constrictive pericarditis post-CABG ^21^	M, 75 years	Two myocardial infarctions	Dyspnea, expectoration	Mediastinal lymph node and pericardial histopathology	Three-vessel coronary disease/CABG	Not specified	Pericardiectomy	Died
Pulmonary TB and unstable angina ^17^	M, 50 years	Hypertension, T2DM, smoker	Chest pain, dyspnea	Apical lung tissue histopathology	Two-vessel coronary disease/CABG	Not specified	Pulmonary mass resection during CABG	Fifteen months of follow-up with good outcome
Latent TB and acute myocardial infarction ^8^	120 patients with latent TB, M: 72 patients (86%), F: 48 patients (14%), mean age: 63 years	Hypertension, T2DM, dyslipidemia, smoker, CKD on RRT	Not reported	QuantiFERON-TB Gold In-Tube test to identify LTBI	Acute myocardial infarction	None	None	None
Coronary arteritis due to TB ^23^	F, 60 years	Hypertension	Chest pain, subcutaneous nodules	Culture and histopathology of subcutaneous nodules	Non-ST elevation myocardial infarction/sequential aneurysms in LAD	HRZE	None	Nine months later without aneurysms in ADA

LAD: Left anterior descending artery. CABG: Coronary Artery Bypass Grafting. T2DM: type 2 diabetes mellitus. CKD: Chronic kidney disease. AFB: acid-fast bacillus. M: Male. HRZE: Isoniazid, rifampicin, pyrazinamide, and ethambutol. F: Female. PCR: Polymerase chain reaction. TB: Tuberculosis. RRT: Renal replacement therapy. LTBI: latent tuberculosis infection.

### Risk factors related to cardiovascular disease and tuberculosis

A study conducted in Uganda on cardiovascular risk factors among people with drug-resistant tuberculosis, which included 212 participants, found that 92% of the population with tuberculosis had ≥ 1 cardiovascular disease risk factor: dyslipidemia (62.5%; 95% CI [confidence interval]: 55.4-69.1), hypertension (40.6%; 95% CI: 33.8-47.9), central obesity (39.3%; 95% CI: 32.9-46.1), smoking (36.3%; 95% CI: 30.1-43.1), elevated body mass index (BMI) (8.0%; 95% CI: 5.0-12.8), and diabetes *mellitus* (6.5%; 95% CI: 3.7-11.1) were highlighted as the main ones ^26^. This interrelation of risk factors could explain the coexistence of adverse cardiovascular events and TB.

Additionally, when reviewing the records of comorbidities found in patients with tuberculosis in Colombia, it is found that 9.7% of the cases had diabetes *mellitus*, 6.4% had chronic obstructive pulmonary disease, and 3.4% had kidney disease ^4^; all of these comorbidities are also related to cardiovascular disease ^27^.

This interrelation of diseases present in both TB and cardiovascular disease could explain what has been reported in the literature: people with TB have a 51% higher risk of experiencing major adverse cardiovascular events ^28^. Consequently, along with the pathophysiological mechanisms that will be discussed later in TB patients, the occurrence of adverse cardiovascular events becomes almost imminent, as does the need for combined treatment. 

### Pathophysiology related to coronary artery disease and tuberculosis

**Figure 1:**
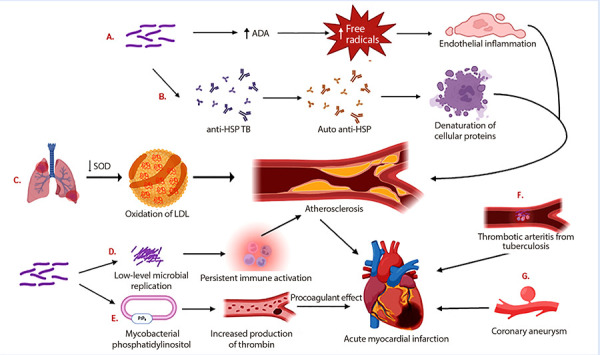
Pathophysiology of the association between coronary disease and tuberculosis.

There is a pathophysiological substrate that reinforces the link between these two entities. Next, we will explain how TB can be part of the genesis of coronary disease and, likewise, how coronary disease, at different stages of its natural history, can be an important predisposing factor for acute manifestations of various forms of TB ^9^^,^^16^^,^^23^; either of these mechanisms may be involved in the initial presentation of our patients (Figure 1).

According to experimental models, the onset of coronary atherosclerosis may be related to endothelial inflammation caused by free radicals secreted by neutrophils stimulated by elevated levels of adenosine deaminase (ADA) in the plasma of patients who have been in contact with tuberculous bacilli ^11^. Additionally, in murine models inoculated with the Bacillus Calmette-Guérin (attenuated strain of *Mycobacterium bovis*), there was a significant increase in aortic atherosclerosis at 8 and 16 weeks without affecting the lipid profile or body weight, suggesting that mycobacterial infections promote the development of atherosclerosis ^12^.

Heat shock proteins, involved in protection against the denaturation of cellular proteins, are targets of immune-mediated reactions by autoantibodies formed through molecular mimicry with mycobacterial heat shock proteins. Elevated levels of autoantibodies have been found in individuals with coronary atherosclerosis and carotid stiffness, having a predictive value for the progression of atherosclerosis ^29^. Additionally, an imbalance between oxidant-antioxidant factors occurs in patients with pulmonary TB, where red blood cell superoxide dismutase (an antioxidant) is decreased, leading to greater oxidation of low-density lipoproteins (LDL) and higher levels of lipid peroxidation, predisposing them to a higher risk of atherosclerosis ^30^.

In patients with latent tuberculosis (LTB), it has been suggested that persistent immune activation related to low-level microbial replication is a driver of coronary disease ^24^. A case-control study found that the presence of LTB is independently associated with acute myocardial infarction (adjusted OR: 1.90; 95% CI: 1.05-3.45) ^24^. Similarly, a systematic review determined that patients with LTB can statistically significantly develop coronary artery disease (OR: 2.15, 95% CI: 1.48-3.12; p <0.0001; I^2^: 0%) ^31^. It is unknown whether treating patients with LTB reduces the incidence of atherosclerosis and consequently cardiovascular disease. Furthermore, it has been suggested that mycobacterial phosphatidylinositol can stimulate thrombin generation and create a procoagulant state ^29^ that leads to acute coronary events ^21^. However, not only can tuberculosis generate both chronic atherosclerotic coronary disease and acute thrombotic coronary events, but post-CABG states can also trigger reactivation of tuberculosis after a latent infection. This is believed to be due to an immunosuppressive state mediated by Interleukin (IL) 6 and IL-10 in the postoperative period ^19^, leading to the reactivation of tuberculosis in various sites such as the sternum ^16^^,^^19^, pericardium ^17^, and pleura ^15^. The risk factors likely related to immunosuppression and consequent reactivation of LTB are the combination of major surgery, cardiopulmonary ischemia, and extracorporeal circulation ^19^.

### Diagnosis of coronary disease and percutaneous intervention

During our search, we did not find specific recommendations for non-invasive diagnosis of coronary disease in patients with tuberculosis. Therefore, we suggest following the recommendations outlined in international guidelines. Regarding coronary reperfusion strategies, data suggest that percutaneous coronary intervention (PCI) and CABG are not contraindicated in patients with acute TB ^32^. Additionally, during CABG, concomitant pulmonary intervention is possible ^33^, which can be useful either for diagnosis or for reducing the tuberculous inoculum. Given the limited information, the safety of these strategies is not clearly established.

### Treatment of ischemic coronary heart disease in patients receiving antituberculosis therapy

Pharmacological management is a cornerstone for the treatment of TB as well as ischemic coronary disease, with or without heart failure, and the drug interactions between them (primarily with rifampicin) are important. Below, we present pharmacological considerations based on pharmacokinetic and pharmacodynamic reports between rifampicin and cardiovascular drugs. 

1. Antiplatelet Agents: Pharmacokinetic and pharmacodynamic studies show that the potent induction of hepatic cytochrome P450 (CYP) by rifampicin does not alter the active metabolite of prasugrel, so no dose adjustment is necessary ^34^. This is not the case with other antiplatelet agents, as rifampicin significantly reduces the plasma concentrations and half-life of ticagrelor (from 8.4 to 2.8 hours) ^35^, while the interaction with clopidogrel enhances its platelet inhibitory effect by increasing its active metabolite ^36^, which could be associated with a higher risk of bleeding. Although these conclusions are drawn from studies in healthy subjects, they indicate that prasugrel is the antiplatelet drug that is not modified by concomitant use of rifampicin. If there are no contraindications, prasugrel would be the suggested drug to use in the context of tuberculosis, followed by clopidogrel in patients with a low risk of bleeding, and subsequently ticagrelor. Acetylsalicylic acid (aspirin) has been used without issues ^(^^20^.

2. Oral Anticoagulants: A study demonstrated that rifampicin increases the clearance of edoxaban by 33% and decreases its half-life by 50% ^37^. For dabigatran, a 67% reduction is observed after one week of co-therapy with rifampicin ^(^^38^. With apixaban, the absolute bioavailability is reduced by approximately 25% ^39^, and with rivaroxaban, there is a 50% reduction in the mean area under the curve (AUC), with a parallel decrease in its pharmacodynamic effects ^40^. Based on these data, the European Heart Rhythm Association recommends avoiding the use of direct oral anticoagulants (DOACs) with rifampicin ^(^^41^. However, a cohort study that evaluated the safety and efficacy of DOACs and warfarin co-administered with rifampicin in patients with atrial fibrillation and venous thromboembolism found, after multivariate adjustment, that the use of DOACs was associated with a lower risk of ischemic cerebrovascular disease (CVD) (HR: 0.51, 95% CI: 0.27-0.94; p=0.030) compared to warfarin, without increasing the risk of major bleeding or death. This risk reduction was found to a lesser extent with warfarin use, suggesting that it may be possible to continue anticoagulation with DOACs in patients with atrial fibrillation and a high risk of CVD ^42^.

3. Lipid-lowering Agents: An in vitro study suggested a possible bactericidal effect of statins on mycobacteria ^43^, although this has not yet translated into clinical benefits ^44^. The concentrations of simvastatin and atorvastatin, along with their active metabolites, are reduced by 43% and 97%, respectively, when administered with rifampicin ^45^^,^^46^. Consequently, it has been advised to increase the total dose of atorvastatin, though the optimal method and potential adverse events remain unclear. In contrast, rosuvastatin has been studied in patients with active TB, demonstrating its tolerance and safety, making it the suggested first-line drug for use in this patient group ^(^^44^..

4. Beta-blockers: These are part of the first-line management in patients with heart failure to reduce mortality and hospitalizations ^47^. In patients with TB, the interaction of these drugs with rifampicin is significant. The pharmacokinetics of metoprolol succinate were evaluated in patients with dialysis and TB, showing a 91% reduction in its levels, becoming undetectable in 75% of patients, leading to adverse clinical outcomes ^48^. The interaction of carvedilol with rifampicin results in a 63% decrease in AUC, while for bisoprolol, the reduction is only 34% ^(^^49^, making bisoprolol the beta-blocker with the least reduction among those approved for heart failure. Therefore, we recommend its use first, in accordance with international guidelines.

5. Angiotensin-Converting Enzyme Inhibitors (ACE Inhibitors), Angiotensin II Receptor Blockers (ARBs), and Sacubitril/Valsartan: ACE inhibitors do not seem to be affected by rifampicin since they are not metabolized by CYP enzymes ^49^. It is worth mentioning that a 31% reduction in the AUC of enalapril’s active metabolite (enalaprilat) has been observed, though this is not entirely clear and does not appear to be secondary to this drug interaction ^49^.

Among ARBs, losartan is a known CYP substrate, resulting in a reduced AUC, and should be avoided as initial therapy. For candesartan and valsartan, the interaction is less well-known, but an increase in their AUC has been suggested, although this is not entirely clear ^49^.

For sacubitril/valsartan, there may be an increase in systemic exposure; therefore, it is recommended to start with low doses and conduct clinical monitoring for adverse events during titration ^(^^50^**6. Diuretics:** Spironolactone and furosemide are unlikely to be affected by rifampicin ^49^, making their use potentially safe since the benefits may outweigh the risks, which remain unclear. No interaction data were found for eplerenone. 

7. Sodium-Glucose Cotransporter-2 Inhibitors (SGLT-2 Inhibitors): Modest reductions in the total exposure to dapagliflozin have been observed, which are not considered clinically relevant ^51^. Similarly, changes in AUC for empagliflozin are minimal ^52^. Therefore, both can be used safely. 

**Figure 2:**
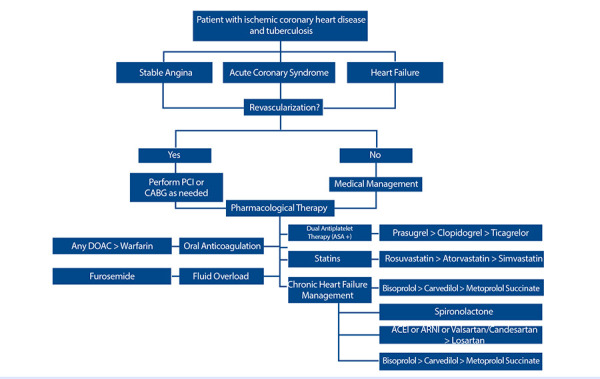
Algorithm for the choice of pharmacological therapy in the management of coronary disease, heart failure, and tuberculosis. The symbol “>” indicates the highest degree of recommendation in descending order from left to right. In the context of non-valvular atrial fibrillation.

In summary, based on the above, we propose an algorithm that synthesizes the management of patients with coronary disease and/or heart failure who have TB (Figure 2). We clarify that the information comes from pharmacokinetic and pharmacodynamic studies, some observational and in healthy patients; however, it is the first approach reported to date for the management of cardiovascular disease coexisting with TB. 

## Conclusions

Ischemic coronary heart disease and TB have a little-known link, forming an unnoticed syndemic that is increasing. Drug interactions related to CYP-mediated metabolism pose a challenge with the use of rifampicin and cardiovascular medications. With this review, we aim to present guidelines for the comprehensive management of ischemic coronary heart disease in the context of tuberculosis to achieve an appropriate approach and reduce complications associated with drug interactions related to these two entities. 
